# The pro- and antineoplastic effects of deoxycholic acid in pancreatic adenocarcinoma cell models

**DOI:** 10.1007/s11033-023-08453-x

**Published:** 2023-05-05

**Authors:** Szandra Schwarcz, Patrik Kovács, Tünde Kovács, Gyula Ujlaki, Petra Nyerges, Karen Uray, Péter Bai, Edit Mikó

**Affiliations:** 1 Department of Medical Chemistry, University of Debrecen, Egyetem Tér 1., Debrecen, 4032 Hungary; 2 MTA-DE Lendület Laboratory of Cellular Metabolism, Debrecen, 4032 Hungary; 3 MTA-DE Cell Biology and Signaling Research Group ELKH, Debrecen, 4032 Hungary; 4 Research Center for Molecular Medicine, Faculty of Medicine, University of Debrecen, Debrecen, 4032 Hungary

**Keywords:** DCA, Pancreatic adenocarcinoma, EMT, Oxidative/nitrosative stress, Stemness, Cell metabolism

## Abstract

**Background:**

Commensal bacteria secrete metabolites that reach distant cancer cells through the circulation and influence cancer behavior. Deoxycholic acid (DCA), a hormone-like metabolite, is a secondary bile acid specifically synthesized by intestinal microbes. DCA may have both pro- and antineoplastic effects in cancers.

**Methods and results:**

The pancreatic adenocarcinoma cell lines, Capan-2 and BxPC-3, were treated with 0.7 µM DCA, which corresponds to the reference concentration of DCA in human serum. DCA influenced the expression of epithelial to mesenchymal transition (EMT)-related genes, significantly decreased the expression level of the mesenchymal markers, transcription factor 7- like 2 (TCF7L2), snail family transcriptional repressor 2 (SLUG), CLAUDIN-1, and increased the expression of the epithelial genes, zona occludens 1 (ZO-1) and E-CADHERIN, as shown by real-time PCR and Western blotting. Consequently, DCA reduced the invasion capacity of pancreatic adenocarcinoma cells in Boyden chamber experiments. DCA induced the protein expression of oxidative/nitrosative stress markers. Moreover, DCA reduced aldehyde dehydrogenase 1 (ALDH1) activity in an Aldefluor assay and ALDH1 protein level, suggesting that DCA reduced stemness in pancreatic adenocarcinoma. In Seahorse experiments, DCA induced all fractions of mitochondrial respiration and glycolytic flux. The ratio of mitochondrial oxidation and glycolysis did not change after DCA treatment, suggesting that cells became hypermetabolic.

**Conclusion:**

DCA induced antineoplastic effects in pancreatic adenocarcinoma cells by inhibiting EMT, reducing cancer stemness, and inducing oxidative/nitrosative stress and procarcinogenic effects such as hypermetabolic bioenergetics.

## Introduction

Pancreatic adenocarcinoma, which has a high mortality rate, is the fourth most common cancer [1]. In 2018, 458,918 cases were reported worldwide and an estimated 432,242 deaths were associated with pancreatic adenocarcinoma [2]. The 5-year survival rate for pancreatic adenocarcinoma is approximately 6% [3]. Since no effective screening method for pancreatic cancer has been developed, most patients are diagnosed at an advanced stage with a poor prognosis [4]. The high mortality rate and aggressive phenotypes of pancreatic cancer are largely due to high metastatic potential.

Dysbiosis refers to a pathological change in the composition of the microbiome. Dysbiosis occurring during neoplasia is termed oncobiosis and the transformed microbiome is the oncobiome [5–7]. The carcinogenic effects induced by oncobiosis support cancer progression and metastasis formation. The oncobiome supports cancer hallmarks, including avoidance of immune destruction, activation of invasion and metastasis, induction of inflammation and angiogenesis, and deregulation of cellular energetics [8–10]. In pancreatic adenocarcinoma, multiple microbial compartments undergo oncobiosis, including oral, gastric, duodenal, fecal, bile, pancreatic duct, pancreatic tissue, and tumor microbiomes [11]. Bacterial dysbiosis may spread from the gastrointestinal tract to the bile duct and pancreatic ducts [12]. Inflammation of the pancreas, due to bacterial infection, is a risk factor for the development of pancreatic adenocarcinoma [13]. Interestingly, fungal colonization of the pancreas is frequently associated with pancreatic adenocarcinoma [14]. Furthermore, after fecal transfer from human pancreatic adenocarcinoma patients, the survival of mice harboring pancreatic adenocarcinoma mirrored the survival of the corresponding patients [15].

In addition to their immunomodulatory role, bacteria can produce metabolites that enter the systemic circulation and exert hormone-like effects [8, 9, 16]. Bacterial metabolites can exert both pro- [17, 18] and anticarcinogenic [19, 20] effects in pancreatic adenocarcinoma. Bacterial metabolites are very diverse in terms of their chemical structure and include secondary bile acids that play a role in carcinogenesis [21]. Secondary bile acids are produced from primary bile acids by deconjugation and dehydroxylation by bacteria [22]. The major secondary bile acids are deoxycholic acid (DCA), lithocholic acid (LCA), and ursodeoxycholic acid (UDCA). Bile acids are largely considered procarcinogenic molecules. However, in previously published studies, bile acids were used in concentrations exceeding the serum or tissue reference range of bile acids, likely resulting in nonspecific effects (reviewed in [21]). High bile acid levels in bile were associated with pancreatic adenocarcinoma [23] and bile acids control multiple steps in carcinogenesis and cancer progression (reviewed in [11]). In previous studies, DCA modulated the cell cycle and procarcinogenic signaling at concentrations higher than the serum reference concentration of DCA in pancreatic adenocarcinoma models [18, 24]. As a model bile acid, we investigated the effects of DCA at the serum reference concentration [8, 25–27] in pancreatic adenocarcinoma cell models to determine the influence of DCA on pancreatic adenocarcinoma behavior.

## Materials and methods

### Cell lines and chemicals

Capan-2 and BxPC-3 cell lines were purchased from the American Type Culture Collection. Capan-2 (human pancreatic adenocarcinoma cells) were maintained in Minimum Essential Medium (MEM, Sigma-Aldrich, St. Louis, MO, USA; cat. no. M8042) containing 10% fetal bovine serum (FBS), 1% penicillin/streptomycin, and 2 mM glutamine at 37 °C with 5% CO_2_. BxPC-3 (human pancreatic adenocarcinoma cells) were cultured in Roswell Park Memorial Institute (RPMI) 1640 medium (Sigma-Aldrich; cat. no. R5886) containing 10% FBS, 1% penicillin/streptomycin, and 2 mM glutamine at 37 °C with 5% CO_2_. Cells were regularly checked for mycoplasma contamination.

DCA (cat. no. B20061) was obtained from Thermo Fisher Scientific (Waltham, MA, USA) and dissolved in dimethyl sulfoxide (DMSO) at a stock concentration of 100 mM. Non-treated cells received vehicle (0.001% DMSO in medium) only. This DMSO concentration corresponds to the DMSO concentration in the DCA-treated cells. The vehicle had no effects on pancreatic adenocarcinoma cell lines.

### Cell invasion assay

Cell invasion assays were performed using Corning BioCoat Matrigel Invasion Chambers with 8.0 µm PET membranes in 24-well plates (Corning, NY, USA; cat. no. 354480). The upper chamber was seeded with Capan-2 cells (20,000 cells/well) in serum-free medium and cultured overnight. Cells were treated with DCA (0.7 µM) in 0.5 ml serum-free medium and the lower chamber was filled with 0.75 ml of serum-containing medium with DCA and 100 ng/ml SDF1-alpha (Sigma-Aldrich; cat. no. SRP4388) as a chemoattractant. After 48 h, invading cells on the lower surface of the membrane were washed in phosphate buffered saline (PBS), fixed with 100% methanol, and stained with 4’,6-diamidino-2-phenylindole (DAPI). The invading cells were counted using an Opera Phoenix High Content Screening System and images were analyzed using the Harmony 4.6 Sofware. The invasion index was calculated from the ratio of invading cells through the Matrigel membrane to cells invading through the control membrane as follows: % Invasion = (Mean of cells invading through Matrigel insert membrane / Mean of cells invading through Control insert membrane) *100 and Invasion index = % Invasion of the Treated cell/% Invasion of the Control (non-treated) cell.

### Real-Time quantitative PCR (RT-qPCR)

Cells were treated with DCA (0.7 µM) or vehicle (DMSO) for 48 h. Total RNA was isolated from cells using TRIzol reagent (Invitrogen, Waltham, MA, USA), according to the manufacturer’s instructions. The RNA samples were treated with DNase (2 μg, Invitrogen). The cDNA was synthesized using a High Capacity cDNA Reverse Transcription Kit (Applied Biosystems, Waltham, MA, USA), according to the manufacturer’s instructions. The qPCR reactions were carried out in a 10 μL volume containing 0.5 μM of each primer and qPCRBIO SyGreen Lo-ROX Supermix (PCR Biosystems Ltd., London, UK). Real-time PCR was performed on the Light-Cycler 480 Detection System (Roche, Basel, Switzerland). The mRNA expression levels for all samples were normalized to the geometric mean of human 36B4 and cyclophilin values. Primer sequences are listed in Table 1.

**Table 1:** Primers used in the RT-qPCR reactions

Gene symbol	Forward primer (5′-3**′**)	Reverse primer (5**′**-3′)
TWIST1	GGGCCGGAGACCTAGATG	TTTCCAAGAAAATCTTTGGCATA
β-CATENIN	TGTTAAATTCTTGGCTATTACGACA	CCACCACTAGCCAGTATGATGA
SNAIL	GCTGCAGGACTCTAATCCAGA	ATCTCCGGAGGTGGGATG
TCF7L2	ACGTACAGCAATGAACACTTCAC	GGCGATAGTGGGTAATACGG
WNT5B	CGGGAGCGAGAGAAGAACT	CGTCTGCCATCTTATACACAGC
36B4	CCATTGAAATCCTGAGTGATGTG	GTCGAACACCTGCTGGATGAC
CYCLOPHILIN	GTCTCCTTTGAGCTGTTTGCAGAC	CTTGCCACCAGTGCCATTATG

### Western blotting

Cells were lysed in RIPA buffer (50 mM Tris, 150 mM NaCl, 0.1% sodium dodecyl sulfate (SDS), 1% TritonX 100, 0.5% sodium deoxycholate, 1 mM ethylenediaminetetraacetic acid (EDTA), 1 mM Na_3_VO_4_, 1 mM NaF, 1 mM phenylmethylsulfonyl fluoride (PMSF), protease inhibitor cocktail). Protein extracts were separated on 10% SDS polyacrylamide gels, transferred to nitrocellulose membranes, and incubated with 5% bovine serum albumin (BSA) to block nonspecific binding sites for 1 h. The blocked membranes were incubated with primary antibodies overnight at 4 °C. After washing, the membranes were probed with IgG horseradish peroxidase (HRP) conjugated secondary antibodies (Cell Signaling Technology, Danvers, MA, USA; 1:2000) for 1 h. Antibody binding was visualized using SuperSignal West Pico Solutions (Thermo Fisher Scientific). Blots were quantified by densitometry using Image Lab 6.1 software. Antibodies sources and dilutions are listed in Table 2.

**Table 2:** Antibodies used in Western blot analyses

Antibody symbol	Vendor	Dilution
β-CATENIN	Cell Signaling Technology (8480)	1:1000
SLUG	Cell Signaling Technology (9585)	1:1000
SNAIL	Cell Signaling Technology (3879)	1:1000
CLAUDIN-1	Cell Signaling Technology (13,255)	1:1000
ZO-1	Cell Signaling Technology (8193)	1:1000
E-CADHERIN	Cell Signaling Technology (3195)	1:1000
iNOS	Novus (NB300-605)	1:1000
4-HNE	Abcam (ab46545)	1:1000
ALDH1	Abcam (ab227948)	1:1000
NITROTYROSINE	Thermo Fisher (A21285)	1:1000
ACTIN	Sigma-Aldrich (A3854)	1:20,000

### Aldefluor assay

Aldehyde dehydrogenase (ALDH) activity in DCA-treated cells was determined using an Aldefluor Stem Cell kit (StemCell Technologies, Vancouver, Canada). Cells were seeded into 6-well plates (100,000 cells/well) and treated with DCA (0.7 µM) for 48 h. Then, cells were incubated in 0.5 ml Aldefluor assay buffer containing ALDH substrate (5 µl/ml) for 45 min at 37 °C. As a negative control, cells were treated with 5 µl of diethylaminobenzaldehyde (DEAB; 50 mmol/l), a specific ALDH inhibitor. The percentage of ALDH-positive cells was determined by flow cytometry and analyzed using Flowing Software 2.5.1.

### Measurement of oxygen consumption and extracellular acidification rate

Oxygen consumption rate (OCR, a readout of mitochondrial oxidation) and changes in pH, termed the extracellular acidification rate (ECAR, readout of glycolytic flux) were measured using a Seahorse XF96 oximeter (Agilent Technologies, Santa Clara, CA, USA). Cells were seeded into 96-well Seahorse assay plates (5000 cells/well) and treated with vehicle or DCA for 48 h before oximetry measurements. Baseline OCR was recorded 5 times for 5 min. Then, cells were treated with etomoxir (50 μM) and OCR was recorded 5 times for 5 min. Next, oligomycin (10 μM) was applied and OCR was recorded 5 times for 5 min. Finally, antimycin (10 μM) was added to the cells and OCR was recorded every 5 times for 5 min. All OCR and ECAR values were normalized to protein content and normalized readings were used for calculations. Fold change values were calculated. Baseline OCR was calculated from basal respiration after subtracting antimycin resistant respiration. Etomoxir resistant OCR (etomoxir-antimycin) refers to the oxygen consumption related to glucose and amino acid oxidation. Etomoxir sensitive OCR (baseline—etomoxir) refers to fatty acid oxidation (FAO). Oligomycin resistant respiration (oligomycin-antimycin) corresponds to uncoupled respiration. Oligomycin sensitive OCR (baseline—oligomycin) refers to ATP production-linked respiration.

### Statistical analysis

Every experiment was independently repeated at least three times. The results are presented as the means ± SEM. Fold data were log2 transformed to achieve normal distribution. Differences between control and DCA-treated groups were evaluated with paired t-tests. Statistical analysis was done using GraphPad Prism 8 software.

## Results

### DCA reduces the expression of epithelial to mesenchymal transition (EMT)-related genes and cell invasion in pancreatic adenocarcinoma cells

**Fig. 1:**
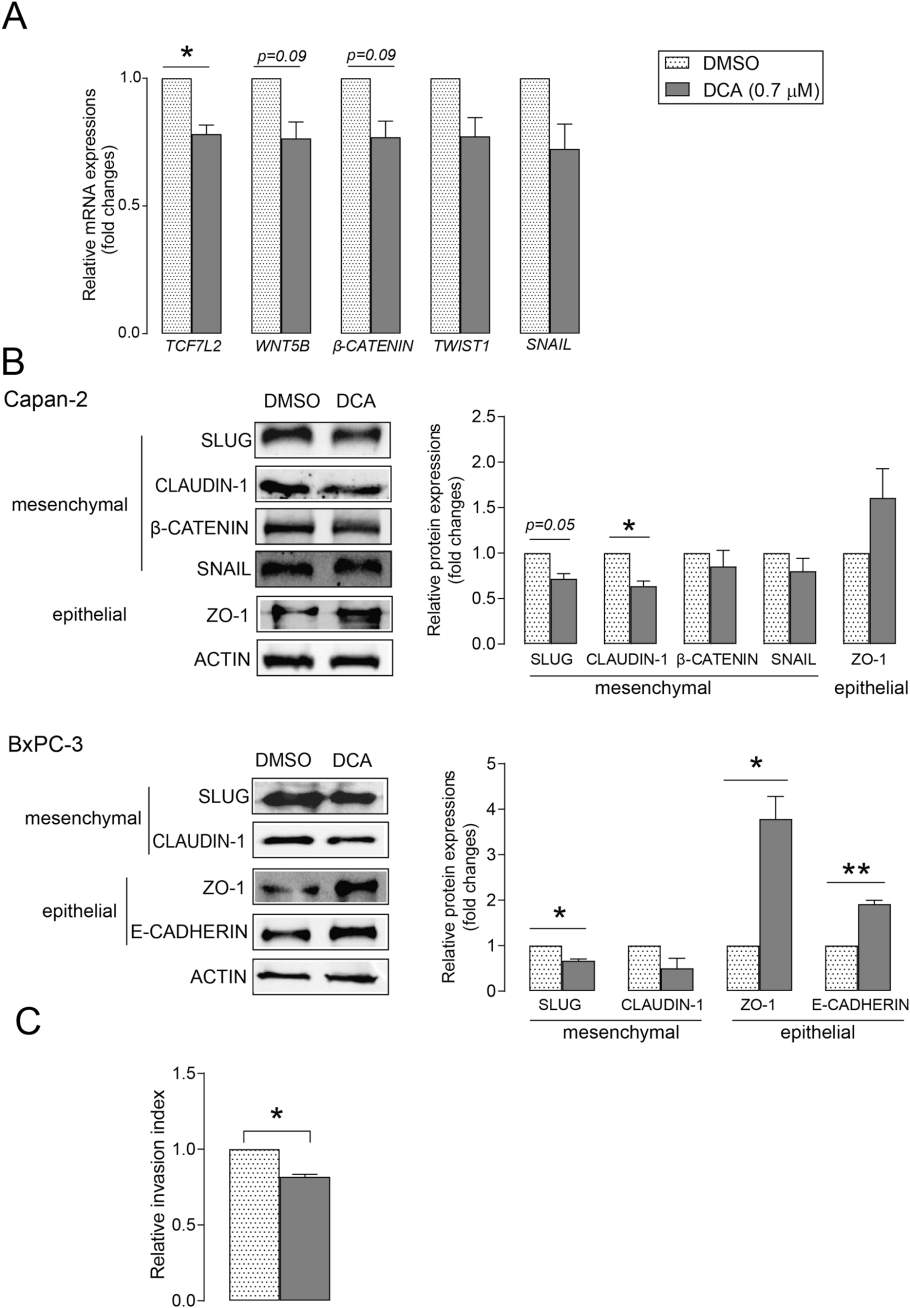
DCA treatment decreases the expression of EMT markers and cell invasion in pancreatic adenocarcinoma cells. **A** After treating Capan-2 cells with DCA (0.7 µM) or DMSO for 48 h, the expression levels of the EMT-related genes, *TCF7L2, WNT5B*, *β-CATENIN, TWIST1, SNAIL,* were determined by RT-qPCR (n = 3). **B** After treating Capan-2 and BxPC-3 cells with DCA (0.7 µM) or DMSO for 48 h, the levels of proteins involved in EMT (mesenchymal: β-CATENIN, SNAIL, SLUG, and CLAUDIN-1; epithelial: ZO-1 and E-CADHERIN) were analyzed by Western blotting (n = 3, left panels: representative figures, right panels: densitometric analyzes of Western blots). **C** Capan-2 cells were treated with 0.7 µM DCA for 48 h and cell invasion capacity was determined using a Corning Matrigel invasion chamber (n = 3). Data are presented as means ± SEM; * and ** indicate statistically significant differences between control (DMSO) and DCA-treated groups at p < 0.05 and p < 0.01, respectively. *DCA* deoxycholic acid; *DMSO* dimethyl sulfoxide; *SLUG* snail family transcriptional repressor 2; *SNAIL* snail family transcriptional repressor 1; *TCF7L2* transcription factor 7-like 2; *TWIST1* twist family BHLH transcription factor 1; *WNT5B* Wnt family member 5B; *ZO-1* zona occludens 1

We assessed whether DCA treatment affected the expression of EMT-related genes. In several studies, bile acids were used in concentrations above the serum or tissue reference range for bile acids, which may have caused nonspecific effects [21]. The DCA concentration used in our study corresponded to the normal concentration of DCA in human serum [8, 25–27]. DCA treatment decreased the mRNA expression of the mesenchymal markers, transcription factor 7- like 2 (*TCF7L2),* Wnt family member 5B* (WNT5B),* twist family BHLH transcription factor 1 (*TWIST1), β-CATENIN,* and snail family transcriptional repressor 1 (*SNAIL)* in Capan-2 cells **(**Fig. 1A**)**. DCA treatment also significantly decreased the protein expression of mesenchymal markers, CLAUDIN-1 in Capan-2 cells and snail family transcriptional repressor 2 (SLUG) expression in the BxPC-3 cell line, whereas DCA induced the protein expression of epithelial markers zona occludens 1 (ZO-1) and E-CADHERIN in BxPC-3 cells **(**Fig. 1B**)**. Cell invasion assays using matrigel invasion chambers demonstrated that DCA significantly reduced cell invasion compared with vehicle treatment **(**Fig. 1C**)**. These results indicate that DCA inhibits EMT through downregulation of EMT-related genes and reduction in cell invasion capacity.

### DCA induces oxidative/nitrosative stress in pancreatic adenocarcinoma cells

**Fig. 2:**
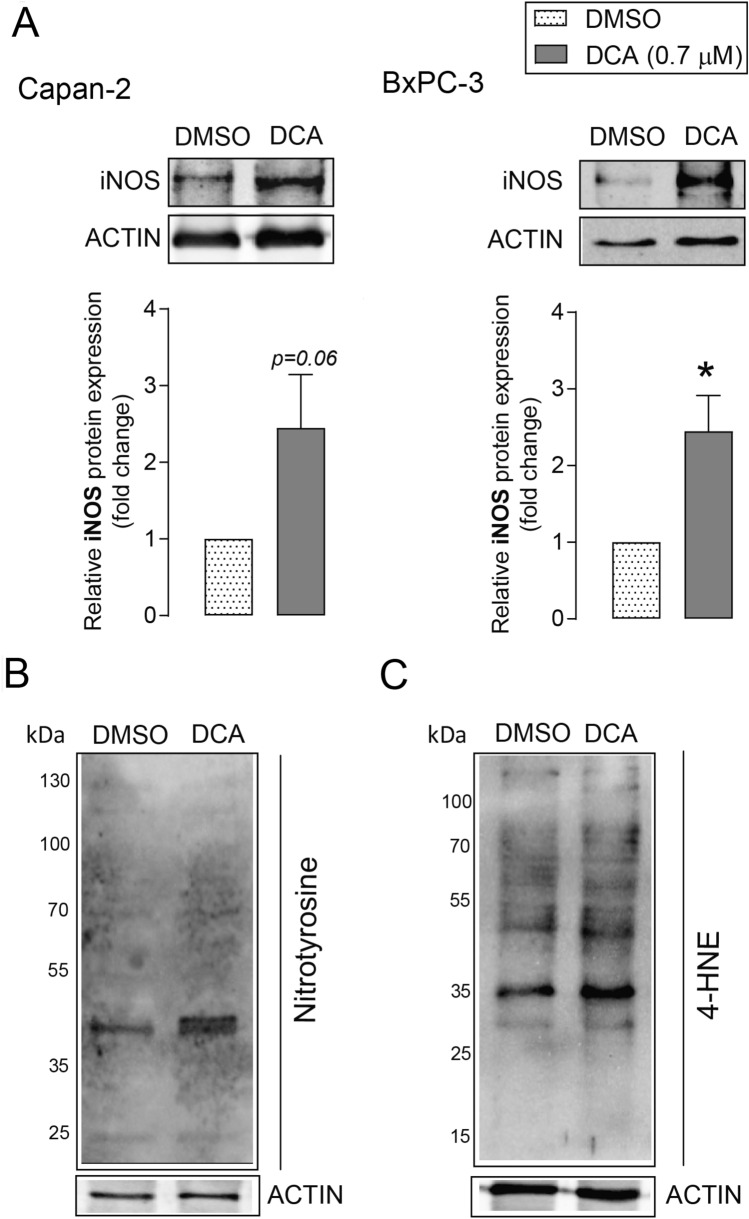
DCA induces the expression of nitrosative/oxidative stress markers in pancreatic adenocarcinoma cells. **A**–**C** Capan-2 and BxPC-3 cells were treated with DCA (0.7 µM) for 48 h. **A** iNOS protein expression was analyzed by Western blotting (n = 4, upper panels: representative western blots, lower panels: densitometric analysis of Western blots from independent experiments). **B** Nitrotyrosine and **C** 4-HNE levels were determined by Western blotting. Similar results were obtained in three independent experiments. Data are plotted as means ± SEM. * indicates p < 0.05, DMSO vs. DCA treated groups. *DCA* deoxycholic acid; *DMSO* dimethyl sulfoxide; *iNOS* inducible nitric oxide synthase; *4-HNE* 4-hydroxynonenal.

We assessed the effects of DCA on the expression of pro-oxidant genes. DCA induced the expression of inducible nitric oxide synthase (iNOS) protein, which generates NO, in Capan-2 and BxPC-3 cell lines **(**Fig. 2A**).** Increased iNOS expression may increase peroxynitrite production, which contributes to enhanced nitrosative stress. Nitrotyrosine levels, indicating peroxynitrite production, increased in DCA-treated Capan-2 cells **(**Fig. 2B**)**. DCA treatment also increased lipid peroxidation, demonstrated by increased 4-hydroxynonenal (4-HNE)-protein adducts in Capan-2 cells **(**Fig. 2C**)**. These results suggest that DCA induces oxidative/nitrosative stress in pancreatic adenocarcinoma cells.

### DCA reduces stemness in pancreatic adenocarcinoma cells

**Fig. 3:**
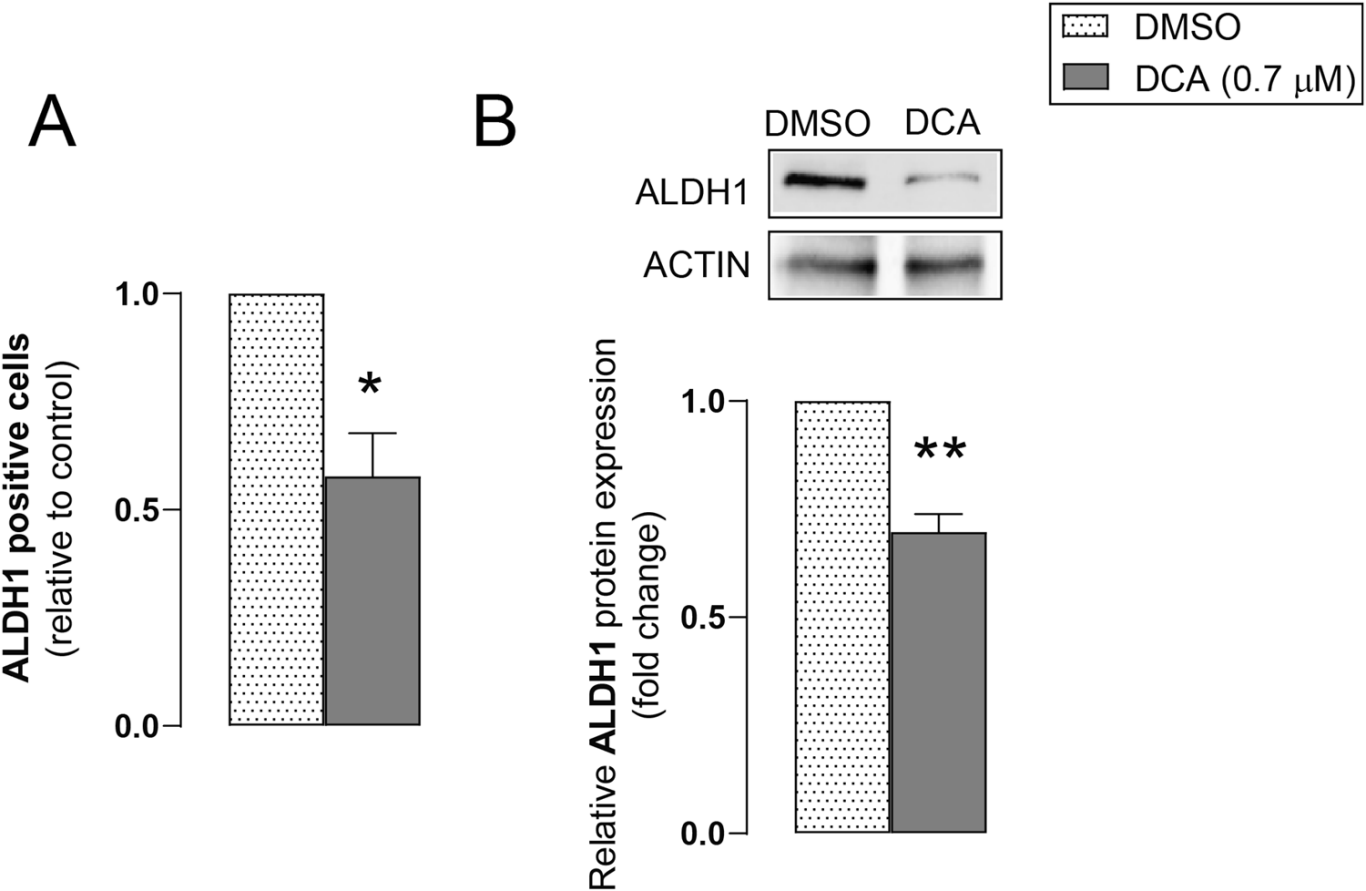
DCA reduces the activity and expression of ALDH1 in pancreatic adenocarcinoma cells. Capan-2 cells were treated with DCA (0.7 µM) or DMSO for 48 h. **A** After DCA treatment, cells were subjected to an Aldefluor assay and ALDH-positive cells were detected using FACS analysis (n = 3 in triplicates). **B** ALDH1 protein was analyzed by Western blotting (n = 3, upper panel: representative figure, lower panel: densitometric analysis of Western blots from independent experiments). Data are presented as means ± SEM. * and ** indicate statistically significant differences between the DMSO and DCA-treated groups at p < 0.05 or p < 0.01, respectively. *DCA* deoxycholic acid; *DMSO* dimethyl sulfoxide; *ALDH1* aldehyde dehydrogenase 1

ALDH1 is a cancer stem cell marker in some tumors [28, 29], including pancreatic adenocarcinoma [30, 31]. We assessed ALDH1 activity after DCA treatment in Capan-2 cells using the aldefluor assay. DCA significantly decreased ALDH-positive Capan-2 cells **(**Fig. 3A**)**. Western blot analyses show that DCA reduced the expression of ALDH1 protein in Capan-2 cells **(**Fig. 3B**)**. These results suggest that DCA reduces stemness in pancreatic adenocarcinoma cells.

### DCA induces mitochondrial activity in pancreatic adenocarcinoma cells

**Fig. 4:**
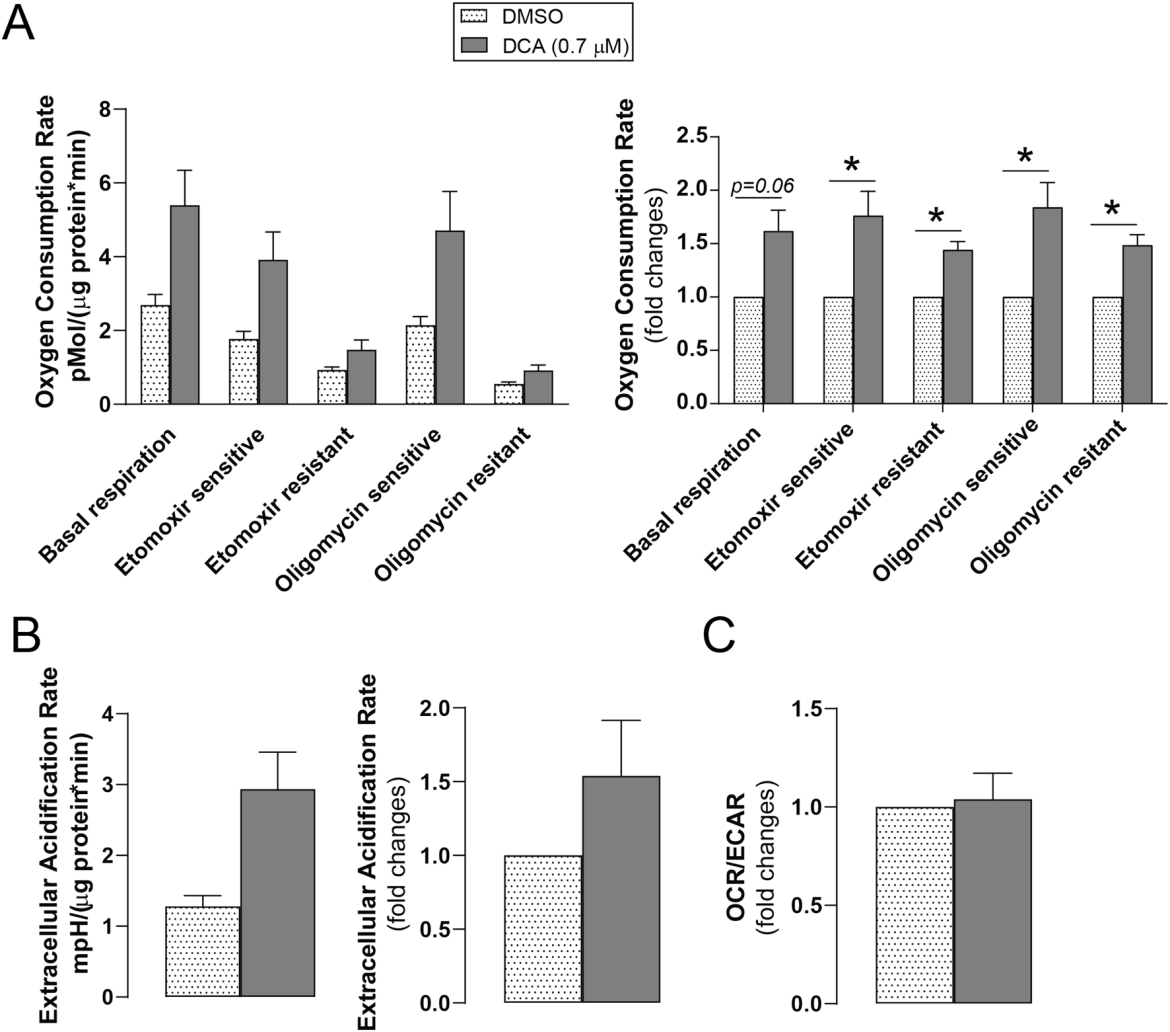
DCA enhances mitochondrial activity in pancreatic adenocarcinoma cells. Capan-2 cells were treated with DCA (0.7 µM) or DMSO for 48 h, and then the rate of mitochondrial oxygen consumption and glycolysis were determined using a Seahorse XF96 analyzer. OCR (**A**) and ECAR (**B**) values were measured (n = 3). The first panel of parts **A** and **B** shows the result of a representative experiment, the second panel shows fold change values of three independent experiments. The OCR/ECAR ratio (**C**) reflects the ratio of mitochondrial oxidation and glycolytic flux. * indicates statistically significant difference between DMSO and DCA-treated cells at p < 0.05. *DCA* deoxycholic acid; *DMSO* dimethyl sulfoxide; *OCR* oxygen consumption rate; *ECAR* extracellular acidification rate

DCA-induced changes in cellular metabolism were assessed. DCA treatment enhanced baseline OCR in Capan-2 cells. Fatty acid oxidation (oxygen consumption blocked by etomoxir termed as etomoxir sensitive respiration) and etomoxir resistant respiration (mostly attributed to glucose and amino acid oxidation) were also enhanced by DCA in Capan-2 cells. DCA significantly increased both oligomycin-sensitive ATP-linked respiration and oligomycin-resistant respiration, reflecting proton leak through the mitochondrial inner membrane **(**Fig. 4A**)**. Moreover, DCA induced glycolysis (ECAR) in Capan-2 cells **(**Fig. 4B**)**. The OCR/ECAR ratio did not change after DCA treatment **(**Fig. 4C**),** indicating that cells become hypermetabolic after DCA treatment.

## Discussion

Bile acid levels, which are elevated in pancreatic adenocarcinoma patients [23], modulate risk factors for pancreatic adenocarcinoma. We demonstrate that DCA treatment inhibits EMT, reduces cancer stemness, induces oxidative/nitrosative stress, and renders cells hypermetabolic. Interestingly, these effects include procarcinogenic features, as well as hypermetabolic bioenergetics and antineoplastic effects demonstrated by the reduced expression of EMT protein and stemness markers and the induced expression of oxidative/nitrosative stress markers.

DCA-induced mitochondrial oxidation, including all fractions of cellular respiration such as oligomycin sensitive and resistant respiration (coupled and uncoupled fraction), etomoxir sensitive and resistant respiration (fatty acid respiration and respiration dependent on other substrates), and glycolytic flux. The ratio of mitochondrial oxidation to glycolysis did not change in response to DCA treatment, suggesting that cells became hypermetabolic but neither glycolysis nor mitochondrial oxidation was dominant. In pancreatic adenocarcinoma, increased mitochondrial oxidation and glycolysis are associated with chemoresistance and cancer cell survival [32, 33]. The relationship between EMT phenotypes and cancer stem cells was investigated in multiple cancer types including pancreatic adenocarcinoma [34, 35]. The reduced expression of EMT and ALDH1 cancer stem cell markers represent DCA-induced antineoplastic features. Finally, DCA induced oxidative (increased 4-HNE production) and nitrosative stress (increased nitrotyrosine production). Overproduction of ROS/RNS species may induce cytostasis or cell death in our cellular models [36]. On a broader perspective, DCA is a bacterial metabolite, and cytostatic bacterial metabolites frequently induce oxidative or nitrosative stress to elicit cytostasis [9].

The effects of DCA on classic cancer hallmarks are different in different cancer cells. For instance, DCA stimulates the proliferation of colon cancer cells [37, 38]. Chen et al. reported [39] that DCA has a malignancy-inducing effect on the transformation of esophageal adenocarcinoma stem cells. DCA activates EGFR, MAPK, and STAT3 signaling through TGR5 receptor and induces tumorigenicity in pancreatic adenocarcinoma cells [18]. DCA promotes migration/invasion in colon cancer [37, 40] and esophageal adenocarcinoma [41] at low concentrations (20 µM), while other studies demonstrated the anti-migratory effects of DCA (100 µM) in gastric cancer [42]. In agreement with this, DCA inhibits gallbladder cancer cell and gastric carcinoma cell proliferation [43, 44]. DCA also induces apoptosis in colorectal [45], ovarian [46], and gastric carcinoma cells [47].

Given the widespread oncobiosis of the human digestive system in pancreatic adenocarcinoma, the value of bacterial metabolite signaling increases as changes to the levels of these metabolites influence the behavior of tumors, even from a distance. We showed that DCA elicits a mixture of pro- and antineoplastic features; hence, the impact of DCA on disease outcome is largely context-dependent. Furthermore, our results show that DCA, and likely other bile acids, retain their biological activity in submicromolar concentrations, although the effects are smaller compared with the effects of high micromolar concentrations demonstrated by previous studies. Taken together, our results show that bile acids modulate the progression and behavior of pancreatic adenocarcinoma and represent an exploitable biological process.

## Conclusion

DCA influences the behavior of pancreatic adenocarcinoma cells by inducing antineoplastic and procarcinogenic effects.

## Data Availability

Primary data is available at https://figshare.com/s/2fa27d1ec6297b74bc76 (https://doi.org/10.6084/m9.figshare.21694706).
